# Supplementary material for: Structural dynamics in the water and proton channels of photosystem II during the S2 to S3 transition
Source: Nat Commun. 2021 Nov 11;12:6531. doi: 10.1038/s41467-021-26781-z (PMC8585918; doi:10.1038/s41467-021-26781-z)
Supplement: Supplementary file 1 — Supplementary Information [file 41467_2021_26781_MOESM1_ESM.pdf]

Supplementary Information for

Structural Dynamics in the Water and Proton Channels of Photosystem II During the S_2 to S_3 Transition

Rana Hussein[1,a], Mohamed Ibrahim[1,a], Asmit Bhowmick[2,a], Philipp S. Simon[2,a], Ruchira Chatterjee[2,a], Louise Lassalle[2], Margaret Doyle[2], Isabel Bogacz[2], In-Sik Kim[2], Mun Hon Cheah[3], Sheraz Gul[2], Casper de Lichtenberg[3,4], Petko Chernev[3], Cindy C. Pham[2], Iris D. Young[2], Sergio Carbajo[5], Franklin D. Fuller[5], Roberto Alonso-Mori[5], Alex Batyuk[5], Kyle D. Sutherlin[2], Aaron S. Brewster[2], Robert Bolotovsky[2], Derek Mendez[2], James M. Holton[2], Nigel W. Moriarty[2], Paul D. Adams[2,6], Uwe Bergmann[7], Nicholas K. Sauter[2], Holger Dobbek[1], Johannes Messinger[3,4*], Athina Zouni[1*], Jan Kern[2*], Vittal K. Yachandra[2*], Junko Yano[2*]

[1]Institut für Biologie, Humboldt-Universität zu Berlin, 10115 Berlin, Germany.

[2]Molecular Biophysics and Integrated Bioimaging Division, Lawrence Berkeley National Laboratory, Berkeley, CA 94720, USA.

[3]Department of Chemistry - Ångström, Molecular Biomimetics, Uppsala University, SE 75120 Uppsala, Sweden.

[4]Department of Chemistry, Umeå University, SE 90187 Umeå, Sweden.

[5]Linac Coherent Light Source, SLAC National Accelerator Laboratory, Menlo Park, CA 94025, USA.

[6]Department of Bioengineering, University of California, Berkeley, CA 94720, USA.

[7]Department of Physics, University of Wisconsin–Madison, Madison, WI 53706, USA.

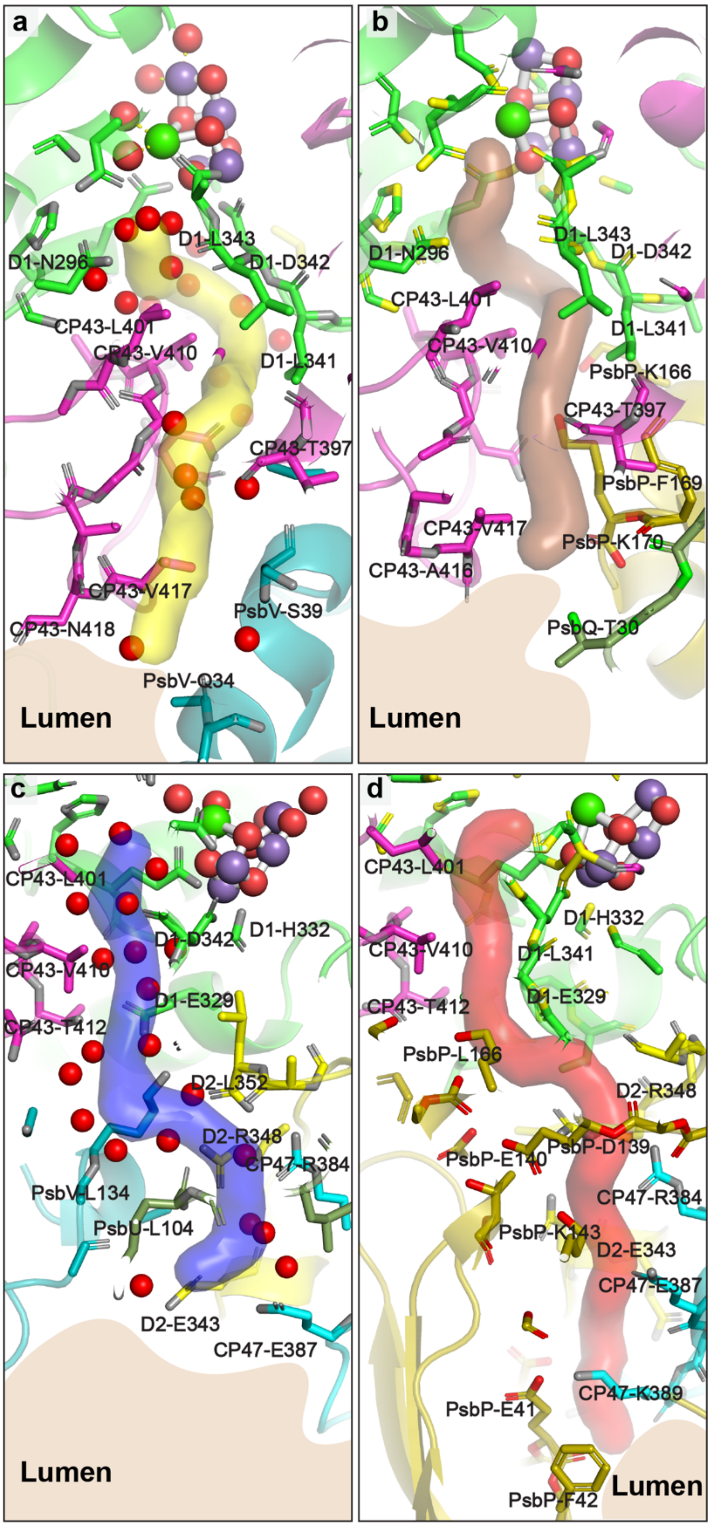

Supplementary Figure 1. Analysis of the O1 channel in cyanobacteria and plants. (a, **c)** represent branches A and B of the O1 channel in the cyanobacterial structure (PDB: 7RF2). (**b**, **d**) show the branches A and B of the O1 channel in the plant PS II structure (PDB: 3JCU). While details at the lumenal termini are different between plants and cyanobacteria due to the different architecture of the lumenal extrinsic part of the complex the region close to the OEC and the general direction and shape are highly conserved for both branches.

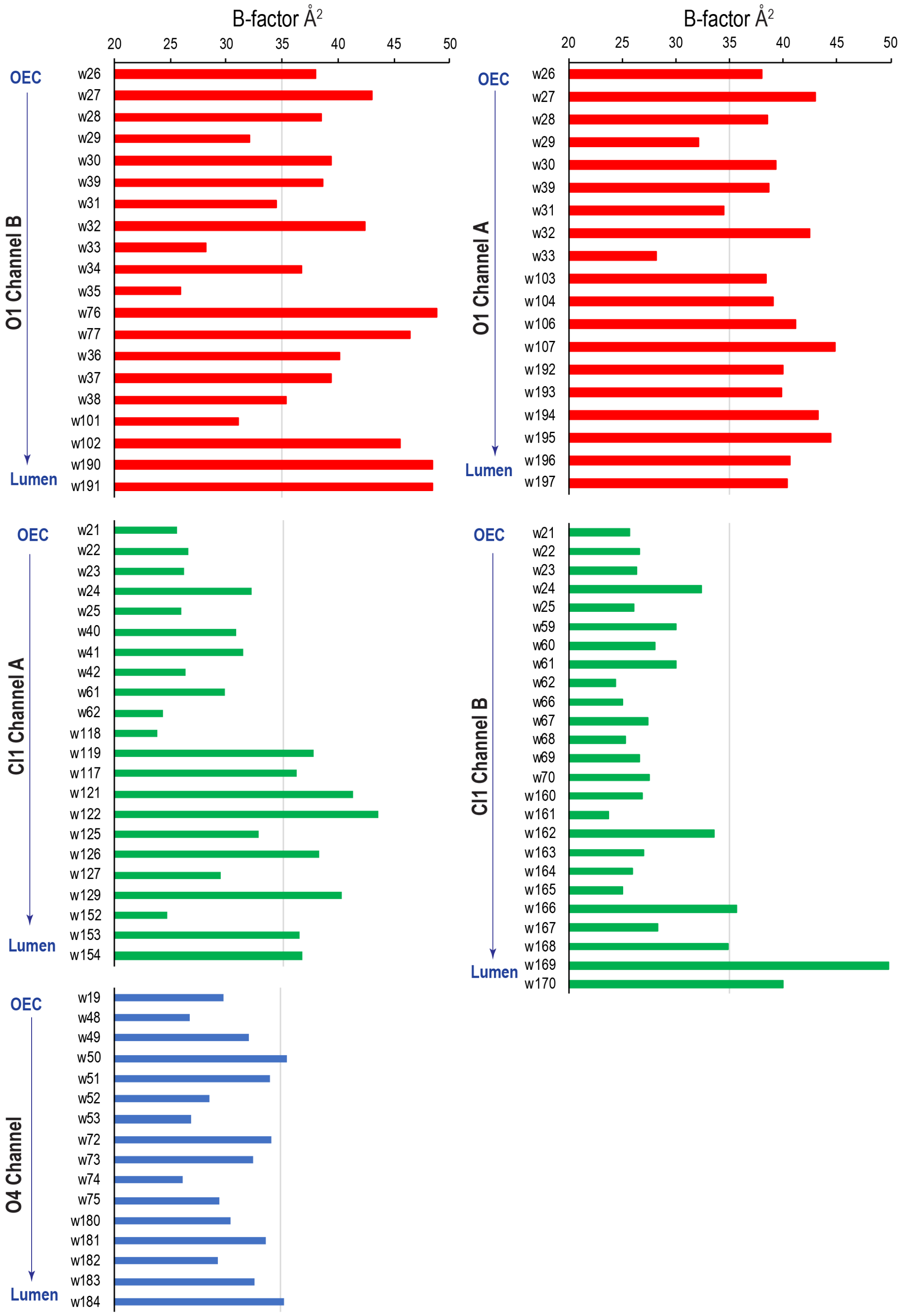

Supplementary Figure 2. The B-factor values of the waters in the O1 channel A, O1 Channel B, Cl1 channel A, Channel B and O4 channel in the combined data (7RF1). Numbering is according to Supplementary Table 4. Source data are provided as a Source Data file.

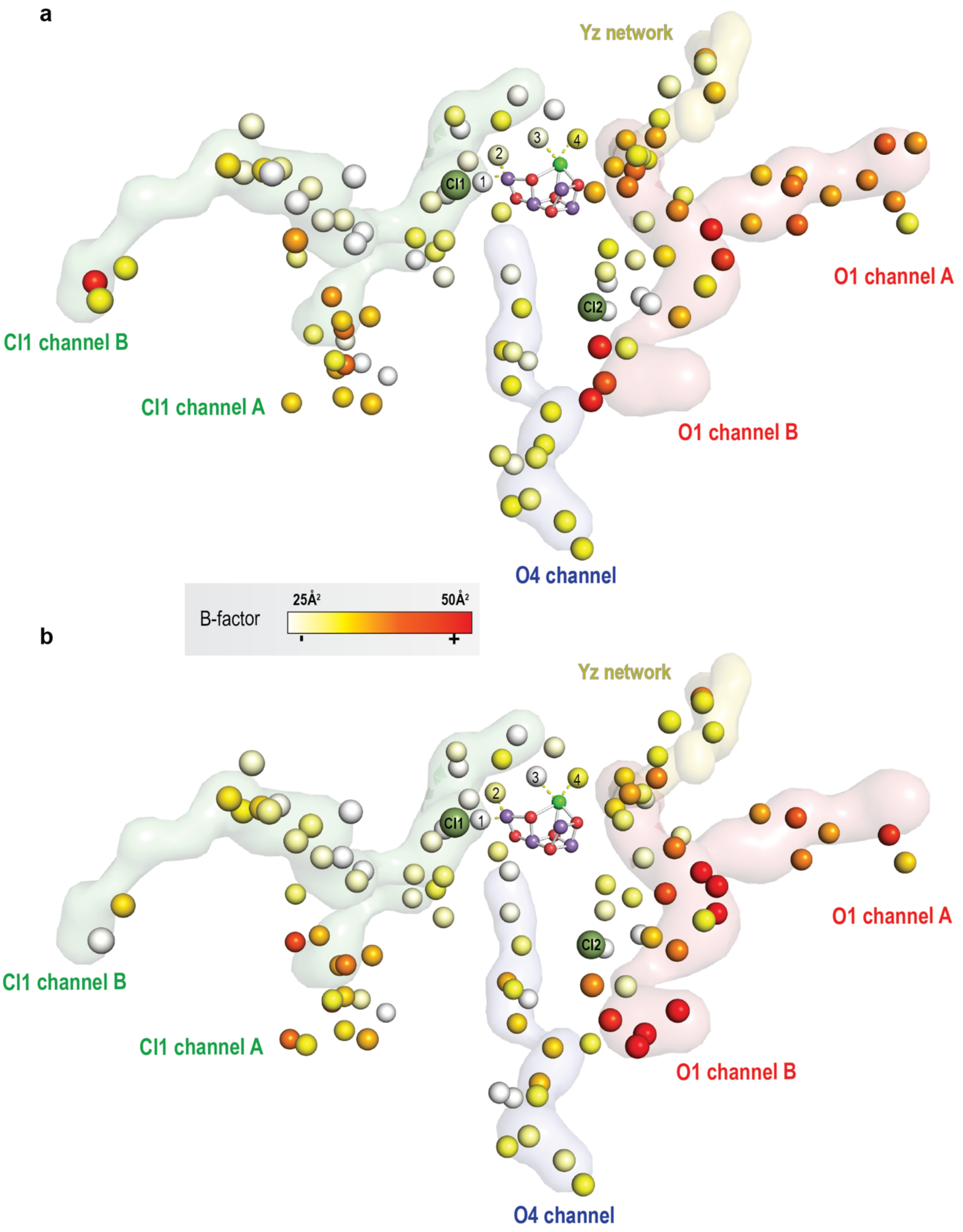

Supplementary Figure 3. Water channels in the 1.89 Å resolution room temperature structure (7RF1) in both monomers m1 (a) and m2 (b). The water molecules are represented in a color gradient scale, indicating the B factor of each water (white color for B-factor 25 to red color for B-factor 50 Å^2 for both monomers). The channels and the network are indicated by different colors, O1 Channel in red, O4 Channel in blue, Cl1 Channel in green, and Yz network in yellow.

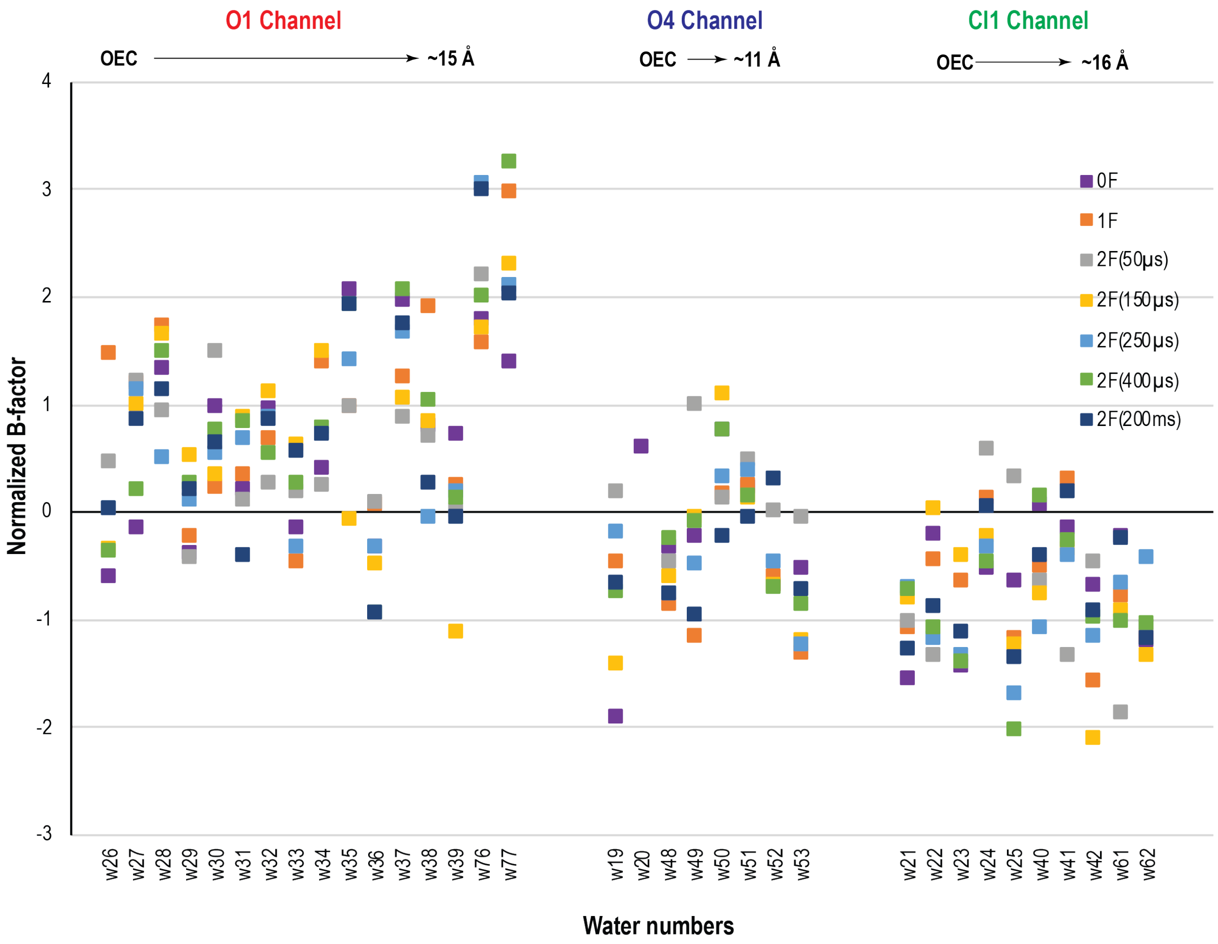

Supplementary Figure 4. Comparison of the normalized B-factors of waters present in the water channels at 0F, 1F, 2F, and the time points between 1F and 2F. The normalized B factors of the waters are shown as a function of their distance from the Mn cluster within the three discussed channels: O1, O4, and Cl1. Source data are provided as a Source Data file.

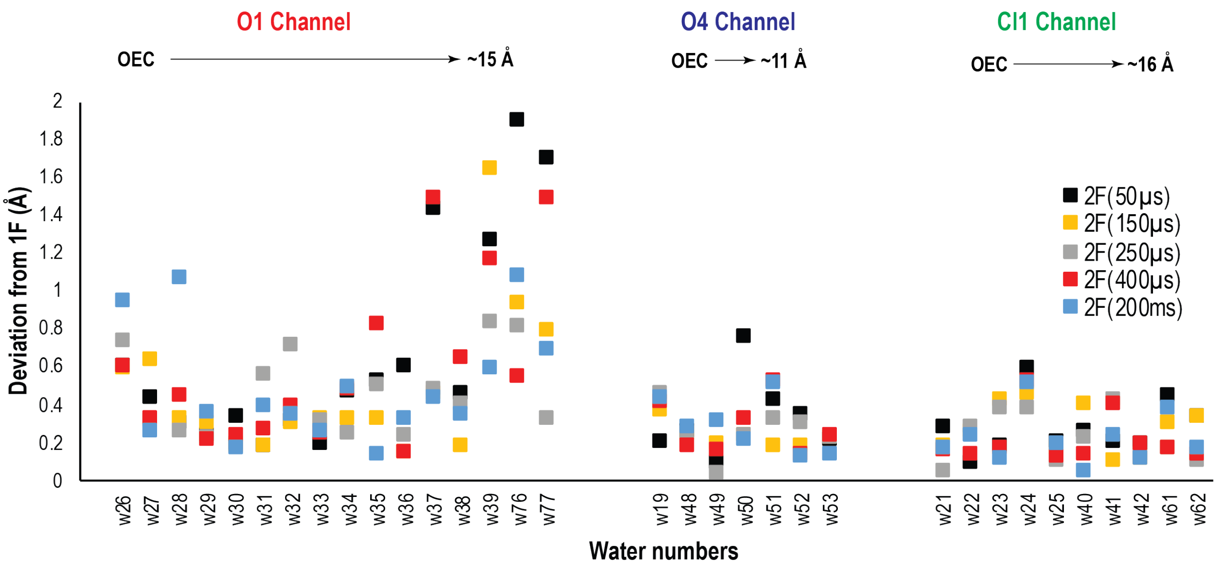

Supplementary Figure 5. The deviation of water positions from the S_2-state (1F) within water channels. The deviation of the water positions for different time points in the S_2-S_3 transition is shown as a function of their distance from the Mn cluster within the three discussed channels: O1, O4, and Cl1. Source data are provided as a Source Data file.

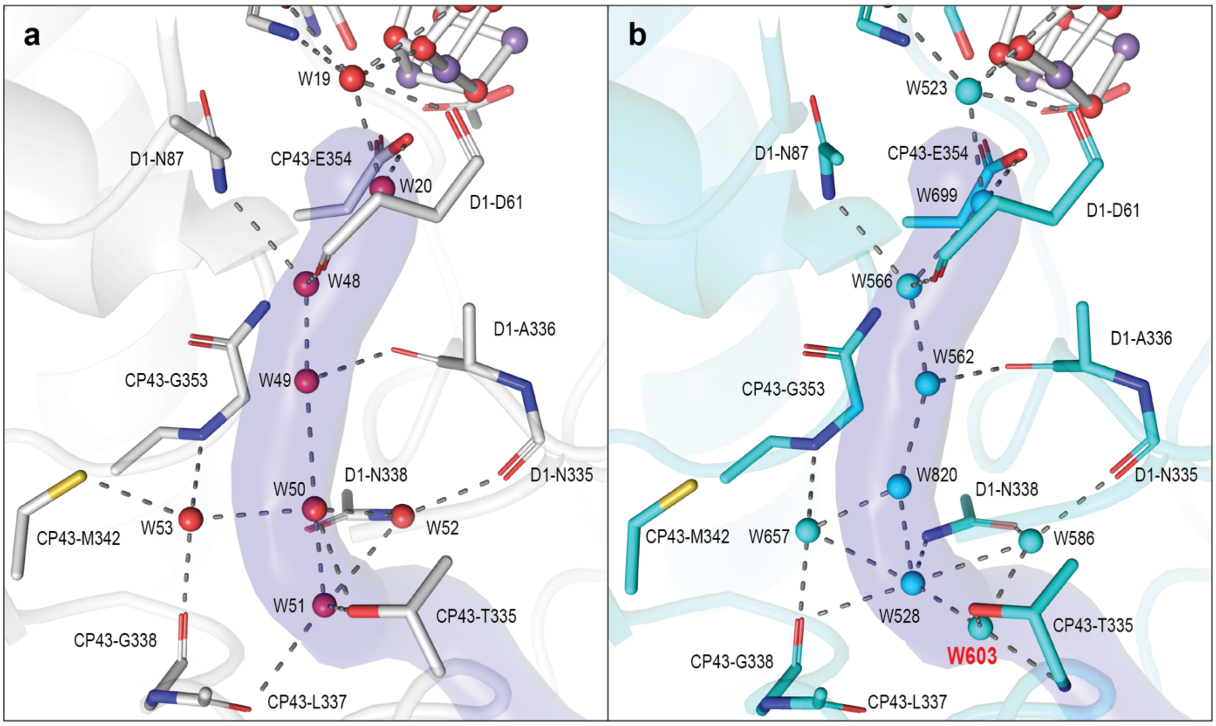

Supplementary Figure 6. Structure of the O4 channel for the dark state at different temperature. Interaction distance between atoms up to 3.2 Å is shown as gray dashed line. The light blue surface represents the O4 channel. A) structure collected at room temperature (PDB ID:7RF2) ref. B) structure collected at cryo temperature with XFEL at 2.15 Å (PDB ID: 6JLJ) [1]. Extra water (W603) (labeled with red color) is detected only in the dark state in the cryo structure effecting the H-bonding networked of the O4 channel.

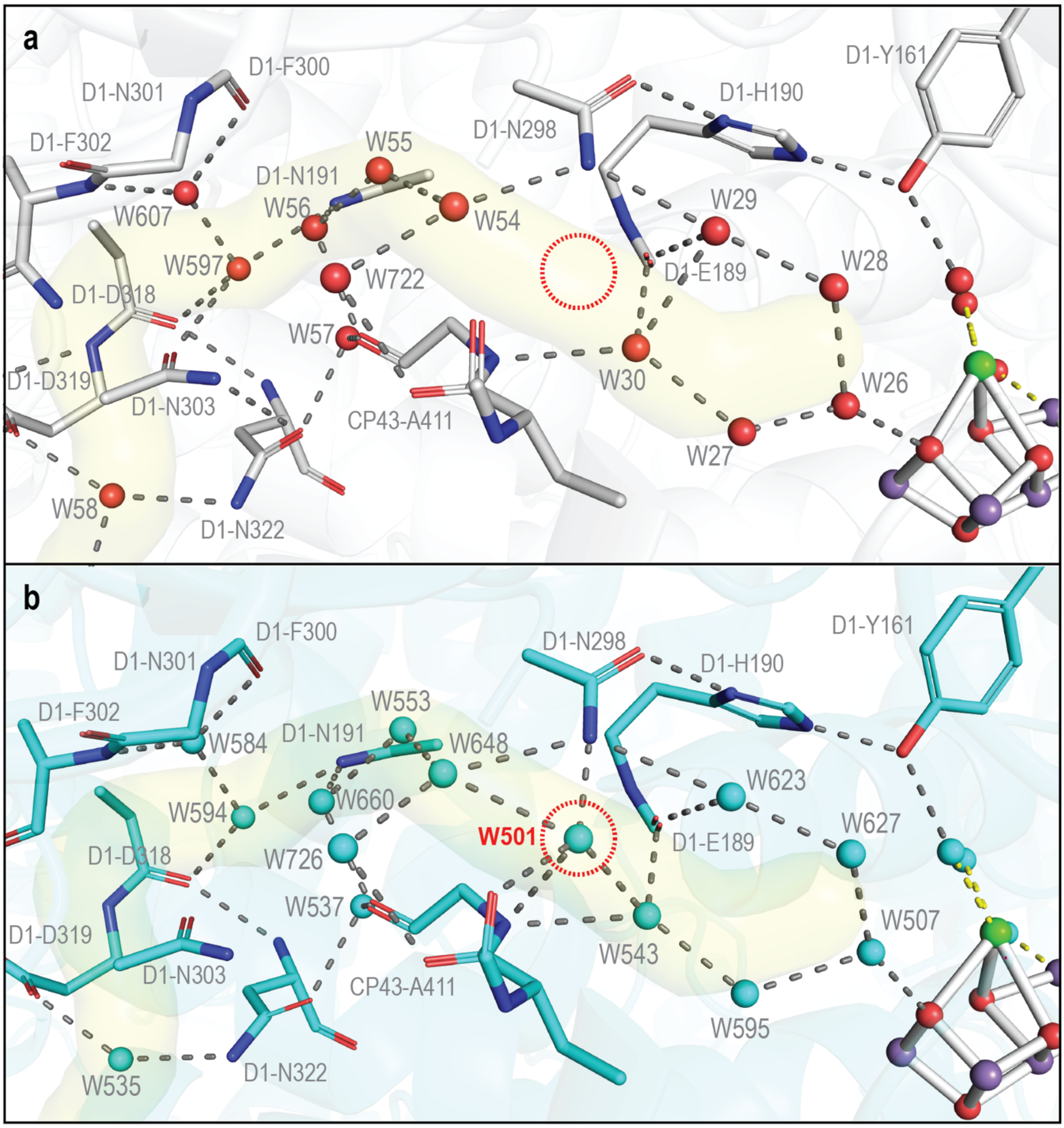

Supplementary Figure 7. Yz network for dark state at different temperatures. Hydrogen-bond distances connecting the Mn_4CaO_5 cluster and Yz toward the luminal bulk side are shown as gray dashed lines and up to 3.2 Å. A) PSII structure with XFEL at RT (PDB ID 7RF2). B) PSII cryo structure with XFEL at 2.15 Å (PDB ID 6JLJ). Extra water (W501) (highlighted with red dashed circle) detected only at the cryo-structure and not in the RT-structure effecting the possible proton path out to the lumen for each structure. In the RT-structure only D1-N298 connects the penta-cluster waters (26-27-28-29-30) via W29 to W54 that proceed towards the lumen. However, in the cryo-structure, in addition to D1-N298, W501 is also connecting the penta cluster waters to the opposite side of the network that expands towards the lumen.

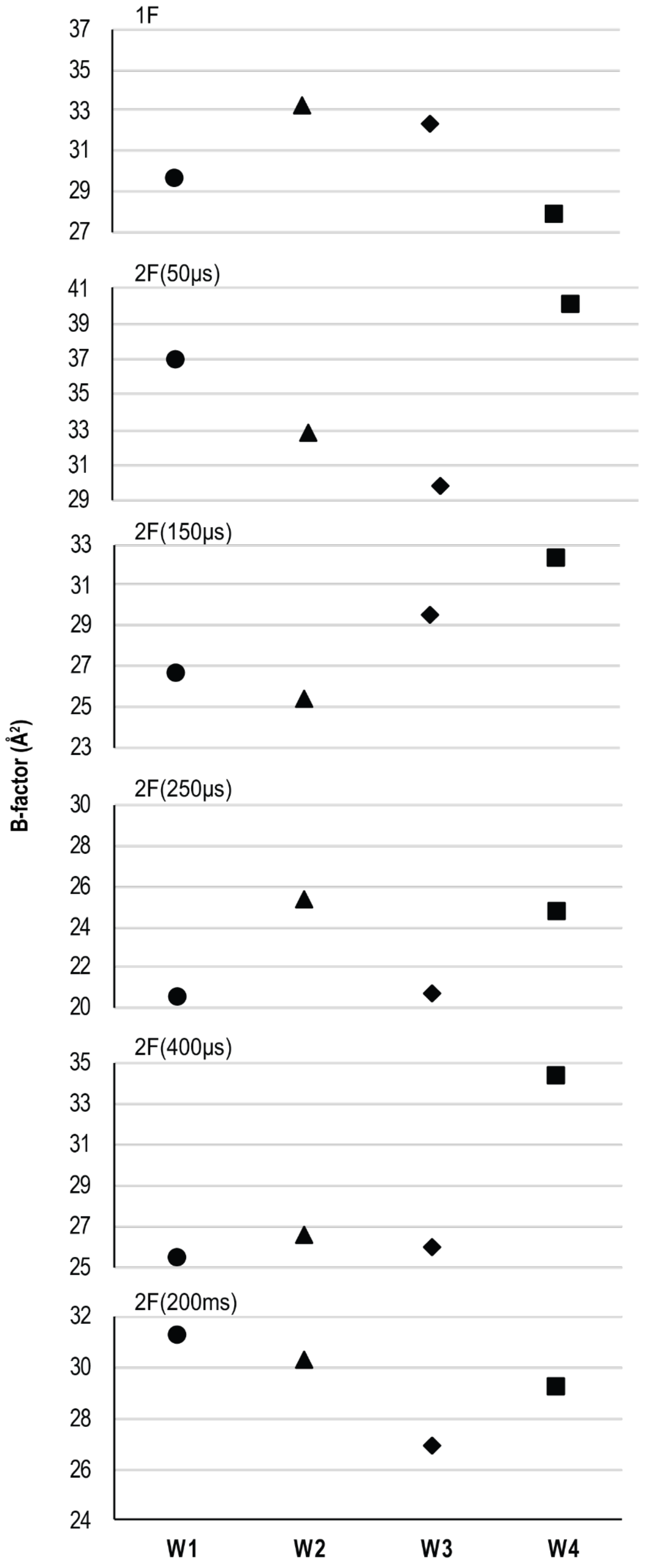

Supplementary Figure 8. B-factors of OEC ligand waters at each time point in the $S_2 \rightarrow S_3$ transition. W4 shows higher B-factor after the second illumination than the other ligand waters (W1, W2 and W3), in the 2F(50µs) structure (PDB ID: 7RF4), the 2F(150 µs) structure (PDB ID: 7RF5), the 2F(250 µs) structure (PDB ID: 7RF6), and the 2F(400 µs) structure (PDB ID: 7RF7). Source data are provided as a Source Data file.

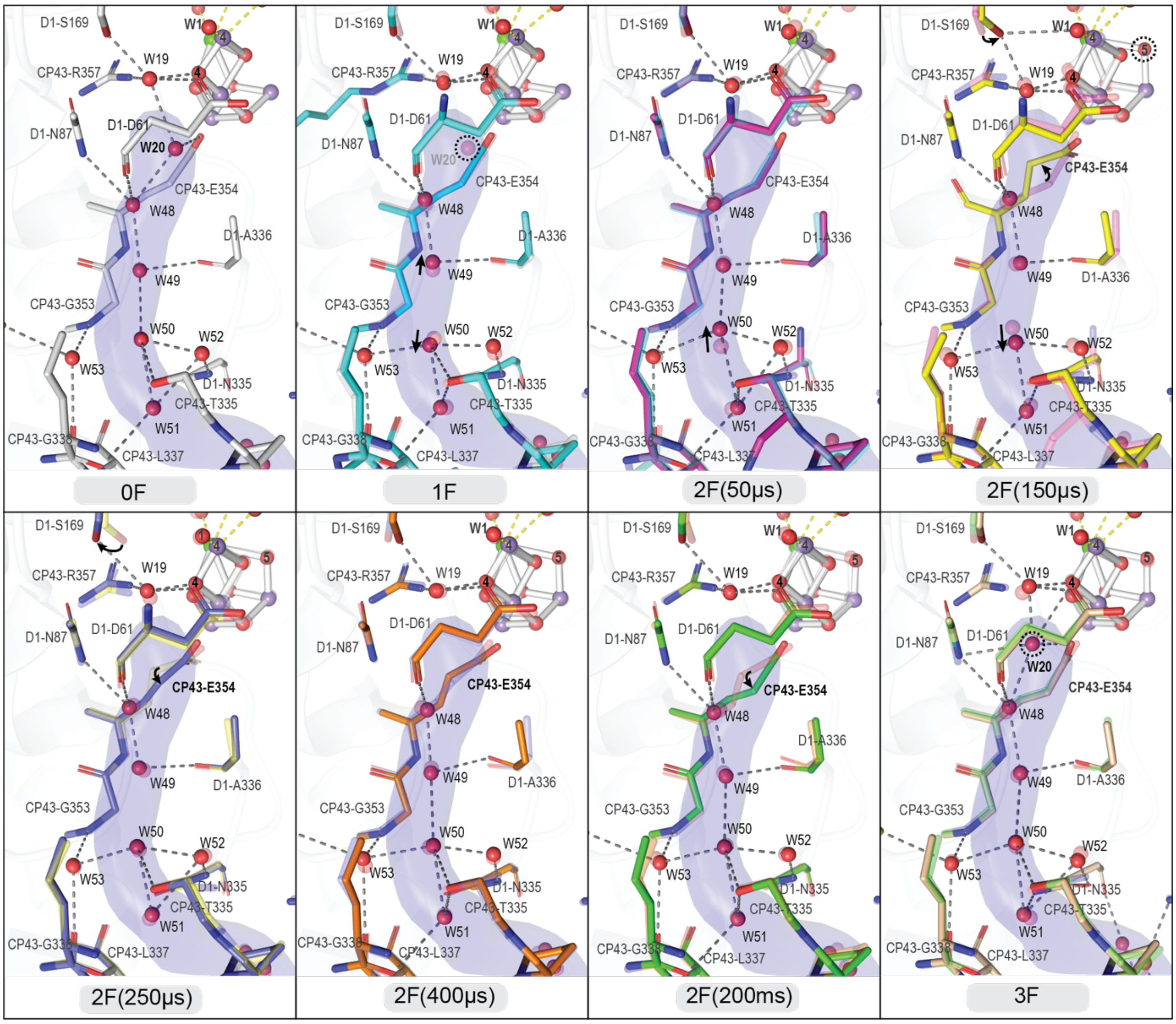

Supplementary Figure 9. Structural changes in the O4 water channel for the different illumination states by XFEL at room temperature. Interaction distances between atoms are shown in gray dashed line and up to 3.2 Å. Positional shifts of the water molecules (W49 and W50) and a.a residues (CP43-E354 and D1-S169) are indicated by black arrows. Structure at 0F is (S_1 state) at 2.08 Å (PDB ID: 7RF2), after 1F (S_2 state) at 2.26 Å (PDB ID: 7RF3), after 2F(50µs) (50µs after S_2 state) at 2.27 Å (PDB ID: 7RF4), after 2F(150 µs) is (150µs after S_2 state) at 2.23 Å (PDB ID: 7RF5), after 2F(250 µs) (250µs after S_2 state) at 2.01 Å (PDB ID: 7RF6), after 2F(400 µs) (400 µs after S_2 state) at 2.09 Å (PDB ID: 7RF7), after 2F (200 µs) (S_3 state) at 2.09 Å (PDB ID: 7RF8), after 3F (S_0 state) at 2.04 Å (PDB ID: 6DHP)[2].

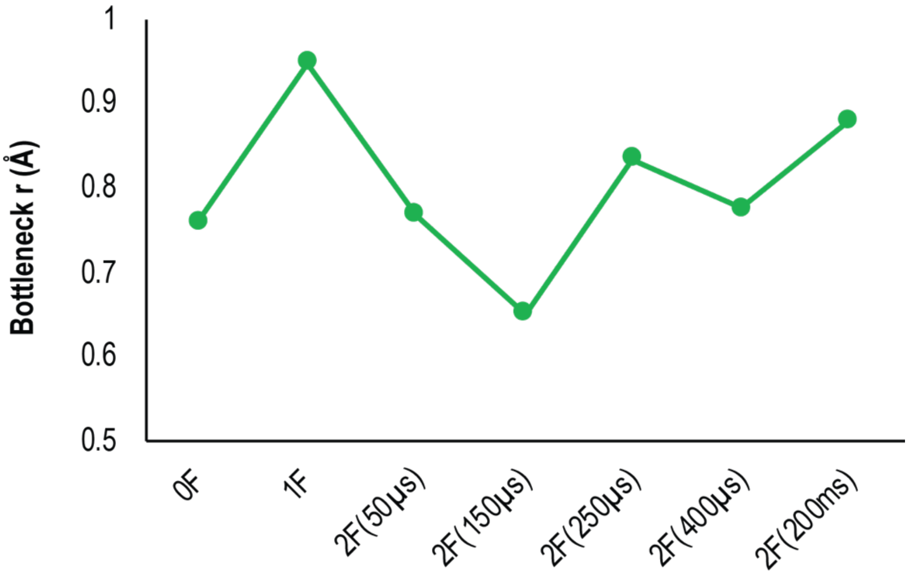

Supplementary Figure 10. Changes in Cl1 channel A bottleneck radius at different time points. Analysis of the bottleneck radius build by D1-E65, D1-P66, D1-V67 and D2-E312 are performed using caver 3.0 Pymol plugin [3] for 0F model (PDB ID: 7RF2), 1F model (PDB ID: 7RF3), 2F(50µs) model (PDB ID: 7RF4), 2F(150 µs) model (PDB ID: 7RF5), 2F(250 µs) model (PDB ID: 7RF6), 2F(400 µs) model (PDB ID: 7RF7), 2F (200 µs) model (PDB ID: 7RF7). Source data are provided as a Source Data file.

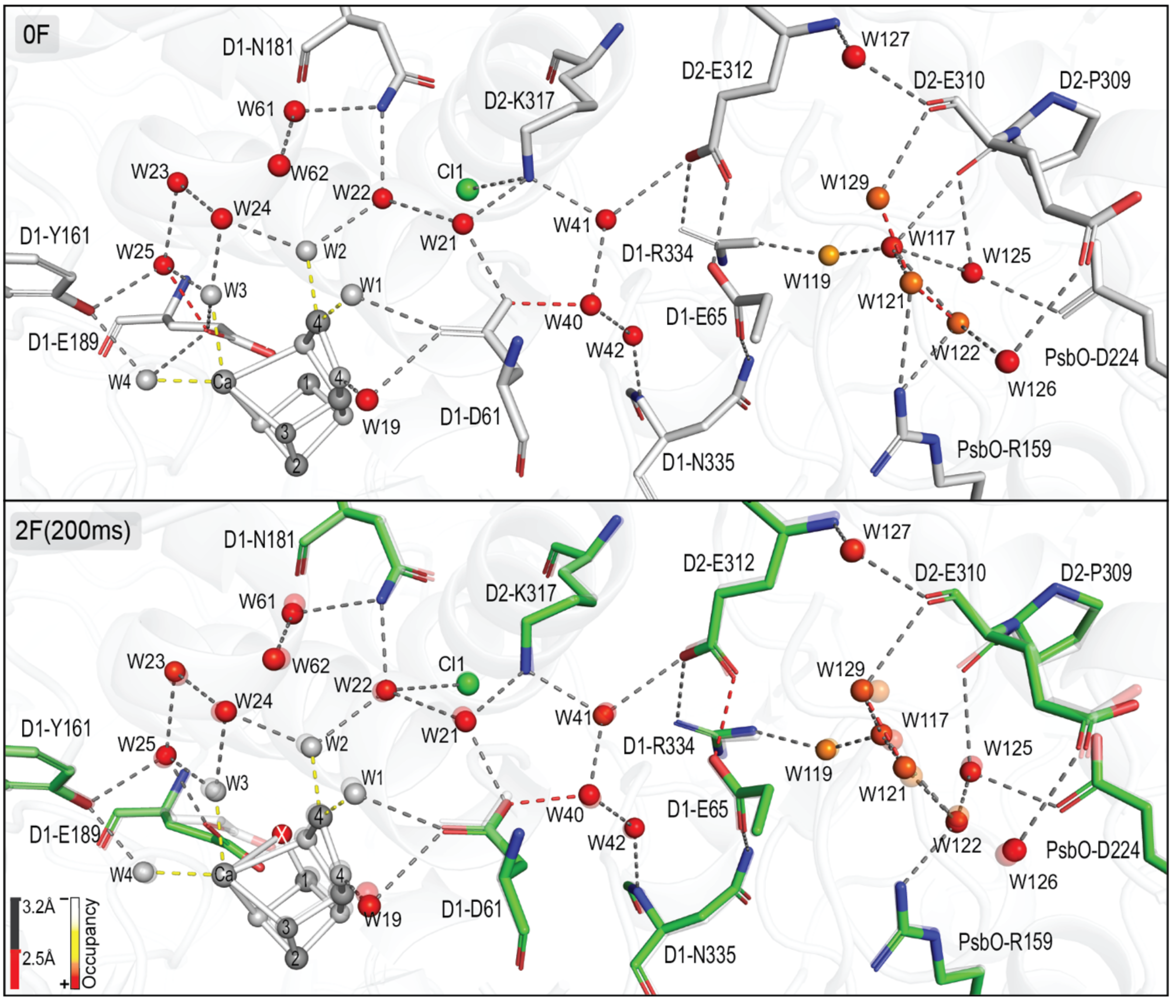

Supplementary Figure 11. Structural changes in the Cl1 water channel detected in the dark state structure (0F, PDB ID: 7RF2) and S3-rich structure (2F (200ms), PDB ID: 7RF8). 0F model is colored with white. 2F (200ms) model is colored with green and overlaid with the 0F model, shown in a transparent color. The waters are colored based on their occupancies, represented by a color gradient from white to red. The H-bond length is color-coded, as described at the bottom left.

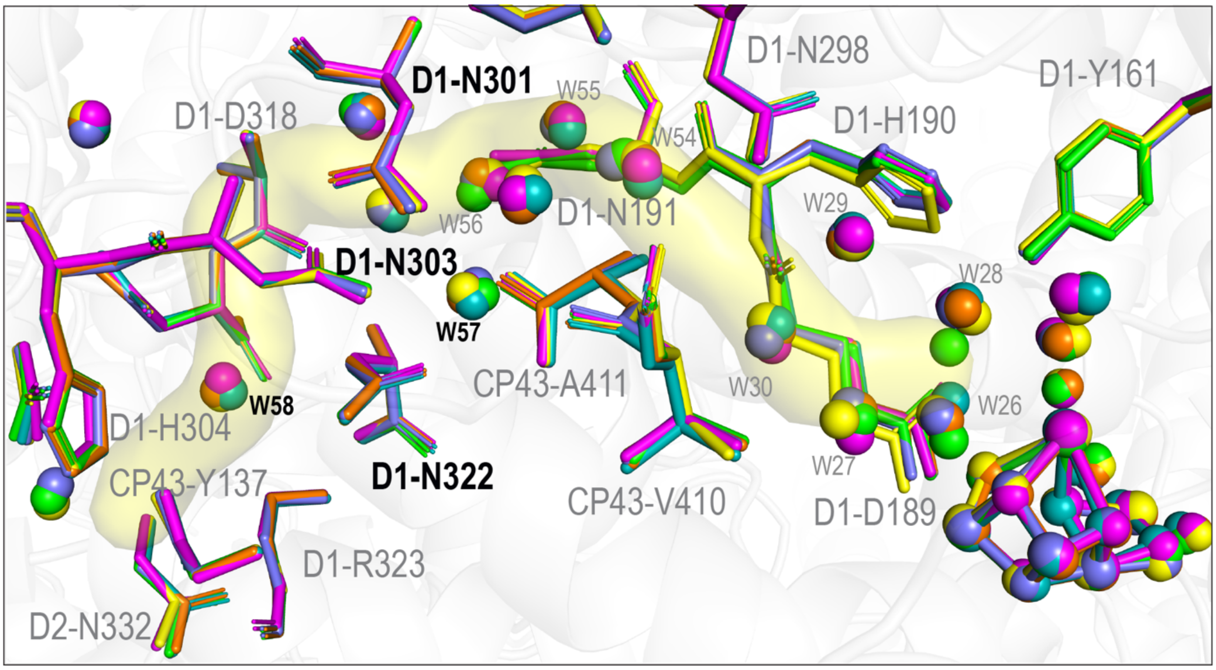

Supplementary Figure 12. Structural changes in the Yz network channel during the $S_2 \rightarrow S_3$ transition. The structure of the Yz network is shown for all time points in the $S_2 \rightarrow S_3$ transition (1F (teal) and 2F time points (50 µs: magenta; 150 µs: yellow; 250 µs: slate; 400 µs: orange; 200 ms: green)).

Supplementary Table 1: Channel nomenclature in literature. There are multiple names used for identifying the water and proton channels in PS II. The table summarizes their correspondence.

	Ho and Styring[4]	Murray and Barber[5]	Gabdulkhakov et al.[6]	Umena et al.[7]	Vassieliev et al.[8]	Ogata et al.[9]	Sakashita et al.[10]	Weisz et al.[11]
Method	Surface contact calculation		Xe	H-bond network analysis	MD simulation			ROS
PDB ID	2AXT		3BZ1	3ARC (2WU2)	3ARC (2WU2)	3ARC (2WU2)	3ARC (2WU2)	
Resolution	3.00 Å		2.90 Å	1.90 Å	1.90 Å	1.90 Å	1.90 Å	
O1 Channel A	large	*channel ii*	B1		4.A		O1-water chain	Arm 2
O1 Channel B	large	*channel ii*	B2		4B			Arm 2
O4 Channel	narrow	NA	E, F	4.c	2	Path 3	O4-water chain	
			D		Channel X			Arm 3
Cl1 Channel A	broad	-	G	4.b	1		E65/E312 channel	
Cl1 Channel B		*channel iii*	C, D		3	Path 2		Arm 1
Cl2 network				4.c				
	back	*channel i*	A1, A2	3.b	5	Path 1		Arm 3

Supplementary Table 2: Merging and refinement statistics

State	Combined	0F[+]	1F[+]	2F (50 μs)[+]	2F (150 μs)[+]	2F (250 μs)[+]	2F (400 μs)[+]	2F[+]
PDB ID	7RF1	7RF2	7RF3	7RF4	7RF5	7RF6	7RF7	7RF8
Resolution range refined (Å)	33.670-1.890	33.638 - 2.080	33.545 - 2.260	33.453 - 2.270	33.698 - 2.230	33.588 - 2.010	33.651 - 2.090	33.649 - 2.090
Resolution range upper bin (Å)	1.923-1.890	2.116 - 2.080	2.299 - 2.260	2.309 - 2.270	2.268 - 2.230	2.0345 - 2.010	2.126 - 2.090	2.216 - 2.090
Wavelength (Å)	1.302	1.302	1.302	1.302	1.302	1.303	1.302	1.302
Space group	$P2_12_12_1$	$P2_12_12_1$	$P2_12_12_1$	$P2_12_12_1$	$P2_12_12_1$	$P2_12_12_1$	$P2_12_12_1$	$P2_12_12_1$
Unit cell parameters (Å)	a=117.0 b=221.6 c=307.7	a=116.9 b=221.6 c=307.8	a=117.0 b=221.6 c=307.9	a=117.1 b=222.1 c=308.4	a=117.0 b=221.8 c=308.2	a=117.0 b=221.9 c=308.3	a=117.0 b=221.7 c=308.2	a=117.0 b=221.6 c=307.8
Lattices merged	111922	11734[+]	4464[+]	5357[+]	6195[+]	8659[+]	5546[+]	10043[+]
Unique reflections	632624	474828	370481	367301	386505	535670	468621	468019
(upper bin)	(31359)	(23014)	(17988)	(17756)	(18749)	(25929)	(22792)	(22666)
Completeness	99.97%	99.77%	99.60%	99.61%	99.64%	99.78%	99.72%	99.75%
(upper bin)	(99.9%)	(97.48%)	(97.47%)	(97.06%)	(97.52%)	(97.34%)	(97.60%)	(97.42%)
$CC_{1/2}$	99.6%	98.0%	96.2%	96.7%	96.5%	97.7%	97.2%	97.9%
(upper bin)	(2.9%)	(5.4%)	(9.1%)	(4.7%)	(5.6%)	(5.1%)	(7.7%)	(4.4%)
$I/\sigma_{Ha14}(I)$[†]	31.2	13.2	11.8	11.8	8.0	14.3	12.3	12.8
(upper bin)	(0.4)	(0.6)	(0.9)	(0.6)	(0.5)	(0.6)	(0.7)	(0.6)
Wilson B-factor	30.3	34.4	37.8	41.3	39.1	32.7	34.4	37.6
R-factor	17.09	18.52	17.82	18.18	17.58	18.02	18.56	18.10
R-free	21.41	23.85	23.79	24.43	23.30	22.70	23.92	23.88
Number of atoms	104289	103675	103283	103257	105567	106133	106087	105942
Number non-hydrogen atoms	52524	52138	51715	51603	52802	53366	53322	53252
Ligands	212	187	192	196	198	195	197	192
Waters	1952	1994	1573	1415	1409	1973	1929	1900
Protein residues	5302	5302	5302	5302	5302	5302	5302	5302
RMS (bonds)	0.01	0.01	0.01	0.01	0.01	0.01	0.01	0.01
RMS (angles)	1.52	1.51	1.45	1.50	1.45	1.43	1.43	1.46
Ramachandran favored	97.3%	97.3%	97.2%	96.5%	96.8%	97.4%	97.4%	97.1%
Ramachandran outliers	0.3%	0.2%	0.2%	0.3%	0.3%	0.1%	0.1%	0.2%
Clashscore	5.7	4.4	5.1	5.7	5.2	4.1	5.3	4.8
Average B-factor	39.0	40.6	43.7	47.2	46.0	39.3	40.1	45.5

[+] Note that the merged datasets for the individual time points are the same as used in ref. 2 but refined models are different.
† as defined in ref. [12]

Supplementary Table 3. Water numbering convention used in this work and relation to residue number*s* in pdb files (note that numbers are only given for the first monomer in each model, “x” indicates water not present, for W1-W4 only the refined component is given for 2F (150 μs) and later time points).

Numbering in manuscript	Channel	Combined (7RF1)	0F (7RF2)	1F (7RF3)	2F (50 μs) (7RF4)	2F (150 μs) (7RF5)	2F (250 μs) (7RF6)	2F (400 μs) (7RF7)	2F (7RF8)
W1		A/515	A/528	A/516	A/526	A/719	A/712	A/518	A/505
W2		A/555	A/579	A/543	A/557	A/728	A/718	A/533	A/537
W3		A/603	A/612	A/582	A/584	A/789	A/803	A/593	A/602
W4		A/525	A/532	A/522	A/513	A/739	A/728	A/564	A/549
W19	O4	A/519	A/526	A/510	A/517	A/715	A/727	A/515	A/529
W20	O4	x	C/675	x	x	x	x	x	x
W21	Cl1A/B	A/512	A/547	A/536	A/569	A/756	A/726	A/514	A/531
W22	Cl1A/B	A/550	A/571	A/557	A/536	A/752	A/729	A/554	A/555
W23	Cl1A/B	A/575	A/588	A/548	A/559	A/791	A/784	A/574	A/603
W24	Cl1A/B	A/622	A/602	A/613	A/581	A/794	A/817	A/616	A/593
W25	Cl1A/B	A/541	A/507	A/540	A/537	A/734	A/713	A/546	A/538
W26	O1A/B	A/612	A/575	A/604	A/566	A/761	A/732	A/552	A/546
W27	O1A/B	A/505	A/508	A/515	A/502	A/710	A/747	A/535	A/508
W28	O1A/B, YZ	A/619	A/601	A/589	A/604	A/775	A/828	A/615	A/625
W29	O1A/B, YZ	A/576	A/613	A/551	A/575	A/764	A/761	A/582	A/600
W30	O1A/B, YZ	A/506	A/527	A/513	A/533	A/714	A/768	A/513	A/509
W31	O1A/B	A/553	A/550	A/528	A/549	A/731	A/704	A/510	A/518
W32	O1A/B	A/637	A/631	A/616	A/614	A/808	A/838	A/638	A/626
W33	O1A/B	A/608	A/595	A/590	A/608	A/777	A/777	A/617	A/611
W34	O1B	A/524	A/524	V/314	A/525	A/732	A/733	A/521	A/517
W35	O1B	C/669	C/618	C/641	C/612	C/664	C/614	C/609	C/608
W36	O1B	D/575	A/577	D/542	A/551	D/512	A/775	D/555	D/564
W37	O1B	U/221	U/211	U/202	U/224	U/209	U/220	U/219	U/204
W38	O1B	V/356	V/351	V/337	V/343	V/341	V/351	V/344	V/359
W39	O1A/B	A/620	A/617	A/599	A/602	A/803	A/748	A/608	A/621
W40	Cl1A	A/534	A/521	A/519	A/528	A/707	A/714	A/529	A/510
W41	Cl1A	D/567	D/527	D/535	D/532	D/519	D/543	D/521	D/550
W42	Cl1A	A/630	A/599	A/612	A/609	A/798	A/833	A/620	A/614
W43	Cl2	A/616	A/585	A/544	A/577	A/788	A/780	A/588	A/587
W44	Cl2	C/764	A/620	A/608	A/599	A/802	A/830	C/725	A/617
W45	Cl2	C/646	C/653	C/664	C/636	C/676	C/682	C/645	C/626
W46	Cl2	C/710	C/660	C/690	C/676	C/679	C/709	C/690	C/695
W47	Cl2	C/692	C/636	C/673	C/629	C/668	C/701	C/669	C/669
W48	O4	A/535	A/581	A/549	A/563	A/782	A/791	A/559	A/558
W49	O4	A/557	A/589	A/574	A/539	A/724	A/781	A/599	A/569
W50	O4	C/723	C/728	C/686	C/694	C/700	C/731	C/702	C/682
W51	O4	C/753	C/750	C/705	C/681	A/797	C/730	C/708	C/710
W52	O4	A/548	A/574	A/545	A/542	A/740	A/703	A/539	A/522
W53	O4	C/696	C/687	C/679	C/678	C/649	C/677	C/665	C/688
W54	YZ	A/623	A/584	C/650	C/635	A/758	C/743	A/609	A/605
W55	YZ	A/521	A/530	A/517	A/512	A/723	A/710	A/508	A/535
W56	YZ	C/631	C/674	C/637	C/608	C/627	C/626	C/649	C/603
W57	YZ	A/504	A/515	A/503	A/504	A/709	A/738	A/502	A/519
W58	YZ	A/590	A/572	A/588	A/532	A/768	A/763	A/571	A/583
W59	Cl1B	A/592	A/605	A/594	A/598	A/793	A/806	A/600	A/571
W60	Cl1B	D/554	D/590	D/556	D/565	D/562	D/553	D/539	D/583
W61	Cl1A/B	A/617	A/614	A/601	A/558	A/795	A/814	A/590	A/601
W62	Cl1A/B	A/510	A/555	A/563	A/544	A/711	A/724	A/534	A/514
W66	Cl1B	D/548	D/541	D/550	D/553	O/335	D/536	D/543	D/532
W67	Cl1B	D/558	O/344	D/562	O/318	O/333	D/540	O/324	O/322
W68	Cl1B	D/608	D/592	D/581	D/586	D/573	D/579	D/558	D/582
W69	Cl1B	D/579	O/330	D/522	D/535	O/309	D/574	D/529	D/519
W70	Cl1B	D/635	D/630	D/600	D/599	D/596	D/614	D/624	D/622
W71	O4	A/511	A/531	A/530	A/543	A/745	U/218	A/579	A/567
W72	O4	D/611	D/609	D/585	D/591	D/591	D/596	D/600	D/608
W73	O4	U/237	U/241	U/225	U/227	U/219	U/230	U/229	U/226
W74	O4	O/326	C/642	O/326	O/320	O/316	O/322	C/652	C/648
W75	O4	U/218	U/216	U/210	U/214	U/213	U/209	U/209	U/213

Supplementary Table 3 continued

Numbering in manuscript	Channel	Combined (7RF1)	0F (7RF2)	1F (7RF3)	2F (50 µs) (7RF4)	2F (150 µs) (7RF5)	2F (250 µs) (7RF6)	2F (400 µs) (7RF7)	2F (7RF8)
W76	O1B	C/670	C/748	C/717	A/612	C/637	C/675	C/628	C/619
W77	O1B	V/363	V/358	V/322	C/685	V/313	V/349	V/363	V/358
W101	O1B	D/573	U/217	D/546	U/219	D/507	D/520	D/536	D/508
W102	O1B	B/863	B/856	D/817	x	x	x	x	B/853
W103	O1A	C/660	C/703	C/625	x	x	C/667	x	x
W104	O1A	C/770	C/759	C/724	C/691	x	C/747	x	x
W106	O1A	C/772	C/761	C/714	C/632	x	C/742	x	C/721
W107	O1A	C/662	C/649	x	x	C/634	C/679	x	C/685
W117	Cl1A	D/623	D/615	x	x	D/578	D/608	D/615	D/615
W119	Cl1A	A/577	A/558	A/504	A/587	A/726	A/757	A/543	A/573
W120	Cl1A	x	x	x	x	x	x	x	x
W121	Cl1A	O/384	O/366	O/353	x	O/357	O/373	O/372	O/382
W122	Cl1A	O/339	O/335	O/337	O/335	O/354	O/318	O/334	O/348
W125	Cl1A	O/348	O/332	O/316	O/333	O/322	O/326	O/332	D/595
W126	Cl1A	O/399	D/617	D/580	D/592	O/364	O/382	D/608	D/605
W127	Cl1A	A/595	D/558	D/528	D/551	D/569	A/794	D/595	D/586
W129	Cl1A	A/556	A/586	A/553	A/592	D/589	A/821	A/613	A/599
W145	Cl1A	x	x	x	O/323	x	x	x	x
W150	Cl1A	x	x	x	x	x	A/786	x	x
W151	O1B	x	x	x	x	x	x	A/516	C/732
152	Cl1A	A/539	A/551	A/552	A/573	A/730	A/754	A/558	A/544
153	Cl1A	O/325	O/349	O/344	x	O/324	O/347	O/329	O/329
154	Cl1A	O/357	O/352	x	x	O/353	O/353	O/354	O/367
160	Cl1B	B/740	B/753	B/771	B/802	B/764	B/740	B/758	B/808
161	Cl1B	D/518	D/534	D/536	D/514	D/527	D/516	D/559	D/522
162	Cl1B	B/769	B/779	B/786	B/807	x	B/796	B/829	B/728
163	Cl1B	D/543	D/545	O/329	O/324	D/523	D/514	D/532	O/331
164	Cl1B	O/336	O/324	x	O/312	O/329	O/314	O/326	O/315
165	Cl1B	B/785	B/746	B/733	B/757	B/762	B/766	B/780	B/764
166	Cl1B	B/821	B/770	B/748	x	B/724	B/752	B/804	B/767
167	Cl1B	D/615	D/598	D/587	D/566	D/590	D/601	D/582	D/585
168	Cl1B	O/319	B/826	O/335	x	B/768	O/325	O/328	O/332
169	Cl1B	B/774	x	x	x	x	x	x	x
170	Cl1B	O/369	x	x	x	O/318	x	x	O/364
180	O4	O/327	O/337	O/330	O/316	O/306	O/312	O/321	O/317
181	O4	U/211	U/220	U/208	U/207	U/204	U/216	U/218	U/208
182	O4	O/404	O/387	O/379	U/229	U/231	O/389	O/379	O/395
183	O4	U/243	U/236	U/232	U/228	U/224	U/239	U/230	U229
184	O4	U/232	U/231	U/223	U/221	U/215	U/225	U/227	U/225
190	O1B	D/528	D/593	x	x	D/517	D/580	D/538	x
191	O1B	D/633	D/628	D/599	x	x	D/613	x	D/626
192	O1A	C/628	x	x	x	x	C/648	x	C/709
193	O1A	C/643	x	C/675	x	x	x	x	C/655
194	O1A	C/625	C/741	C/684	C/649	x	x	C/616	C/720
195	O1A	C/714	C/713	x	V/312	x	x	x	V/311
196	O1A	C/762	x	x	x	x	x	x	C/721
197	O1A	C/728	C/708	x	C/638	x	C/686	C/678	C/687

Supplementary Table 4. The peak heights of individually generated polder-omit maps for waters present in the water channels and up to approximately 15 Å from the OEC at 0F, 1F, 2F and the time points between 1F and 2F in both monomers (m1) and (m2). The waters with 3-5 σ are depicted in bold.

m1

W	0F	1F	2F50	2F150	2F250	2F400	2F
19	12.02	9.9	8.26	10.2	9.83	10.51	9.02
20	7.67	--	--	--	--	--	--
21	10.83	10.56	8.6	8.75	11.49	10.11	10.24
22	9.58	9.21	9.3	7.45	10.78	10.55	8.91
23	10.79	9.9	10.01	8.49	10.84	10.06	9.26
24	9.49	8.92	7.44	7.97	9.12	8.55	8.76
25	9.00	8.98	5.61	9.15	11.63	11.5	10.31
26	9.5	6.9	6.95	7.62	8.17	7.45	7.44
27	8.85	6.24	5.63	4.73	6.73	7.91	6.75
28	7.61	7.21	6.6	6.74	8.47	6.69	6.89
29	9.5	10.01	8.22	7.91	9.23	8.42	8.16
30	7.8	6.71	4.74	6.73	7.21	6.65	6.23
31	7.88	7.66	7.34	6.88	6.58	6.34	7.76
32	6.85	7.77	7.2	6.48	6.28	6.65	6.25
33	9.1	8.56	8.33	7.58	9.2	8.07	7.67
34	8.03	6.55	6.75	5.78	7.58	7.1	7.09
35	6.46	6.98	6.76	7.48	5.8	6.72	6.67
36	9.53	8.63	7.87	9.05	8.6	9.87	9.86
37	6.63	6.81	6.33	7.57	6.25	5.66	6.49
38	7.05	6.36	6.49	6.97	8.23	6.41	7.23
39	4.59	**3.61**	5.1	5.71	4.82	5.67	5.13
40	9.08	8.19	8.13	8.19	10.4	8.37	8.64
41	9.47	8.04	9.36	7.94	8.94	8.93	8.46
42	9.69	11.02	9.26	10.08	11.1	10.59	9.85
43	10.15	9.32	9.74	8.5	10.6	8.47	10.4
44	8.79	7.84	7.68	6.72	9.17	8.12	8.36
45	9.3	9.9	11.08	10.32	10.62	10.29	9.99
46	10.36	9.98	8.04	10.79	10.65	9.7	10.11
47	10.41	11.41	8.55	9.77	11.03	10.86	10.57
48	10.13	10.44	8.45	9.35	10.77	9.23	9.42
49	8.89	9	5.8	7.78	8.5	7.97	9.18
50	8.05	8.28	7.11	7.41	7.84	7.6	8.17
51	8.13	8.99	7.18	8.29	8.32	8.71	8
52	9.28	9.07	7.67	8.77	9.75	9.94	8.64
53	8.81	9.96	7.57	9.9	10.48	9.61	8.9
54	9.46	8.07	8.09	8.85	8.96	9.3	8.38
55	7.57	7.73	7.03	7.71	8.39	8.86	9.07
56	6.59	7.67	6.68	7.28	8.81	7.79	7.67
57	8.67	6.73	7.78	6.09	8.94	7.8	7.14
58	8.81	8.99	9.26	9.34	11.45	9.06	9.19
59	8.85	8.85	8.99	7.57	9.83	9.28	8.12
60	8.48	8.22	8.02	9.72	9.47	9.6	8.31
61	9.35	9.29	9.59	8.81	10.58	10.34	9.48
62	10.29	10.43	8.37	9.64	9.73	9.25	9.38
63	9.88	8.61	6.77	7.93	10.01	6.94	9.55
64	8.65	9.03	6.78	8.82	9.5	8.16	8.61
65	8.3	7.66	7.54	7.3	6.99	8.02	7.61
66	10.96	10.78	10.61	10.23	11.88	10.95	10.11
67	11.36	9.45	9.29	11.45	10.98	10.79	10.68
68	10.71	10.92	9.59	9.73	11.07	10	10.74
69	10.82	10.65	10	9.22	11.96	11.77	10.39
70	10.49	8.9	8.2	10.68	12.31	10.65	10.21
71	7.01	7.34	5.9	7.02	9.01	8.15	8.22
72	8.1	9.06	5.37	8.7	8.73	8.72	7.55
73	10.24	9.4	8.89	9.32	10.35	10.21	9.3
74	10.29	10.05	10.63	10.67	11.61	10.09	10.41
75	10.29	9.84	8.87	8.95	11.52	10.03	8.51
76	6.12	6.44	5.4	6.6	5.13	6.41	**3.28**
77	6.05	**4.74**	5.19	**4.13**	5.5	6.06	5.58

m2

W	0F	1F	2F50	2F150	2F250	2F400	2F
19	10.61	8.06	7.8	8.7	9.68	9.27	9.17
20	6.75	--	--	--	--	--	--
21	9.78	10.81	10.35	9.96	10.83	10.61	10.72
22	9.23	10.23	10.41	9.19	10.06	10.64	10.62
23	9.77	8.97	7.23	8.75	9.8	9.5	9.12
24	9.89	9.88	8.74	9	9.32	9.17	9.66
25	8.79	9.23	8.95	9.53	10.17	10.32	9.01
26	8.86	8.56	8.12	8.99	8.96	8.26	7.29
27	6.82	5.6	7.12	4.08	5.03	4.2	6.14
28	7.77	7.24	6.8	9.11	8.71	7.98	6.85
29	8.97	8.42	8.23	8.48	8.56	9.15	9.14
30	7.74	7.6	8.45	7.26	7.19	7.14	8.22
31	6.5	6.85	5.93	6.32	7.85	6.84	7.61
32	7.68	8.35	6.28	6.47	8.37	6.25	6.28
33	9.8	8.29	9.13	9.82	9.17	8.55	9.9
34	6.34	7.66	5.71	7.52	7.07	7.5	7.89
35	7.84	8.24	7.06	7.96	6.42	6.94	6.96
36	8	7.95	6.64	8.78	9.04	8.32	7.52
37	5.62	6.67	5.32	6.02	7.76	7.82	6.1
38	8.7	7.88	6.64	9.01	9.58	8.73	7.44
39	--	--	--	**4.34**	--	**4.16**	5.33
40	9.79	8.83	6.83	7.34	8.87	9.14	8.85
41	8.85	10.99	8.46	8.44	10.32	10.39	10.07
42	8	8.82	8.69	8.44	9.27	9.3	9.19
43	8.33	6.85	7.06	8.54	8.82	8.52	9.49
44	8.95	7.09	8.78	7.7	8.5	7.72	8.16
45	8.47	9.32	10.34	10.13	9.76	9.09	9.84
46	11.07	9.17	9.64	10.39	11.44	10.32	10.77
47	8.94	9.93	9.07	9.59	10.85	10.73	9.95
48	11.83	9.7	10.66	10.29	9.84	10.24	10.09
49	9.37	10	8.22	9	9.33	9.72	8.33
50	9.28	8.01	7.98	9.33	8.7	7.51	7.42
51	7.92	8.42	8.35	7.7	8.14	8.51	7.42
52	8.91	7.87	7.96	9.49	9.93	8.65	8.62
53	9.24	9.5	9.2	10.46	9.72	10.87	9.97
54	8.78	8.88	9.68	9.42	9.71	8.65	8.84
55	8.84	7.85	8.2	8.61	8.35	7.72	7.84
56	7.55	8.63	9.08	8.34	9.31	8.54	9.02
57	8.99	7.74	8.11	7.84	8.22	8.05	8.77
58	10.1	8.27	7.63	8.66	9.25	8.51	9
59	9.49	9.62	7.72	8.57	8.54	7.47	9.73
60	10.21	10.56	9.03	10.4	9.43	9.58	10.64
61	9.96	8.63	6.34	8.56	8.85	9.74	10.18
62	10.25	8.58	9.39	9.37	10.81	11.76	10.5
63	8.6	9.31	8.08	8.82	10.06	10.49	10.07
64	9.23	8.42	7.76	9.06	10.24	9.07	9.37
65	8.57	8.01	7.87	7.67	8.52	8.75	7.72
66	10.96	11.4	9.36	10.06	12.25	11.8	11.7
67	10.48	10.51	9.28	9.51	11.21	10.95	11.22
68	9.01	9.51	8.17	9.08	10.97	9.23	9.49
69	9.14	8.72	7.84	9.79	10.3	10.15	9.17
70	9.76	8.55	9.02	9.58	10.18	9.9	10.32
71	8.24	10.13	7.47	7.47	9.02	8.08	9.59
72	7.06	8.4	7.23	8.08	9.23	8.23	8.7
73	8.37	8.56	6.96	9.04	8.26	8.18	8.24
74	8.56	9.04	8.1	8.91	9.19	8.47	9.85
75	9.67	10.22	8.33	9.34	10.81	8.76	9.2
76	**4.72**	6.42	5.65	5.86	**4.77**	5.33	6.43
77	5.11	6.64	5.38	5.51	5.16	5.64	5.34

Supplementary Table 5. The B-factor (Å^2) of waters present in the water channels and up to approximately 15 Å from the OEC at 0F, 1F, 2F and the time points between 1F and 2F in both monomers (m1) and (m2).

				m1								m2			
W	0F	1F	2F50	2F150	2F250	2F400	2F	W	0F	1F	2F50	2F150	2F250	2F400	2F
19	19.9	32.5	41.0	28.7	26.8	27.0	37.5	19.0	21.9	33.9	32.0	30.1	22.6	27.9	25.8
20	33.0	--	--	--	--	--	--	20.0	27.2	--	--	--	--	--	--
21	22.9	27.5	32.4	33.7	26.3	30.9	34.4	21.0	21.9	24.2	26.9	30.0	21.9	24.8	24.7
22	29.2	30.2	30.4	28.9	25.6	26.0	31.6	22.0	29.4	26.5	26.6	32.4	25.9	25.0	27.3
23	26.9	32.7	33.7	32.3	25.3	25.1	33.7	23.0	22.7	26.2	32.6	32.6	24.7	24.9	24.7
24	30.4	35.8	36.4	31.9	28.1	27.6	31.6	24.0	26.2	27.5	40.6	35.1	29.4	27.0	31.9
25	60.5	28.7	36.5	30.7	22.5	21.8	27.1	25.0	29.3	28.1	33.9	31.5	22.3	29.0	34.6
26	30.2	36.8	40.0	32.4	26.6	27.5	29.4	26.0	33.1	35.8	35.3	30.3	25.5	30.5	32.5
27	31.9	37.1	39.3	32.1	29.6	30.3	34.3	27.0	34.0	34.6	39.4	34.3	31.4	33.5	34.1
28	36.6	32.6	41.1	34.0	27.1	31.8	38.4	28.0	35.6	34.2	37.5	32.3	26.1	28.3	34.2
29	32.0	32.9	38.2	37.2	31.5	33.2	39.4	29.0	34.9	37.5	37.7	35.7	27.8	30.1	35.3
30	36.1	39.0	48.5	39.4	33.8	34.2	40.0	30.0	40.9	37.3	41.0	39.5	32.3	33.2	43.0
31	37.8	39.6	40.4	37.3	32.2	32.9	37.5	31.0	39.7	37.0	39.9	37.1	30.3	36.1	36.9
32	37.3	40.3	38.1	36.2	32.7	31.3	37.9	32.0	36.8	36.1	42.4	40.9	29.1	33.4	38.1
33	29.9	35.6	38.1	39.3	29.5	35.5	38.8	33.0	29.0	36.2	31.8	33.3	34.0	34.7	35.0
34	32.0	39.3	40.0	42.0	36.8	35.6	39.8	34.0	43.8	43.1	46.7	45.9	42.3	42.0	44.5
35	41.1	39.4	45.2	36.0	36.2	38.3	46.5	35.0	37.5	39.6	41.2	39.1	39.5	38.1	44.5
36	28.7	35.6	40.0	32.5	30.5	29.2	30.7	36.0	33.3	37.8	43.1	36.3	35.3	33.2	36.8
37	37.7	39.4	42.5	38.4	35.9	38.4	42.8	37.0	45.2	41.3	45.2	43.7	39.8	37.4	45.0
38	32.7	39.3	43.0	37.6	29.9	32.0	40.4	38.0	29.2	36.5	42.7	36.6	32.0	33.5	40.7
39	36.8	35.5	36.5	34.9	27.0	31.9	37.1	39.0	--	--	--	32.1	--	30.1	39.4
40	31.9	30.4	36.3	29.4	22.1	34.9	28.9	40.0	24.8	31.0	40.3	34.9	27.1	27.0	31.8
41	28.7	33.7	30.8	30.4	30.0	31.3	32.2	41.0	31.4	26.8	31.9	32.2	25.6	23.6	28.0
42	27.5	24.7	36.9	27.1	25.1	27.8	26.1	42.0	28.7	32.6	28.1	28.3	25.0	27.0	24.2
43	24.8	30.1	30.0	36.9	27.0	31.5	30.7	43.0	27.5	30.9	33.9	29.4	28.1	31.8	28.5
44	30.3	37.3	37.8	36.3	25.5	30.8	37.2	44.0	22.6	29.4	30.0	33.1	20.8	27.2	26.7
45	27.3	25.6	24.6	23.4	25.8	25.9	30.4	45.0	29.9	27.9	25.0	28.1	29.4	29.5	26.3
46	22.2	25.0	32.0	25.8	24.7	25.5	33.1	46.0	16.3	31.2	30.4	26.9	24.3	28.9	23.4
47	20.7	20.2	34.9	29.5	23.5	23.8	27.4	47.0	27.5	26.3	30.2	29.1	25.2	23.3	27.5
48	28.9	29.4	35.5	35.8	27.5	32.2	30.8	48.0	18.7	27.6	23.1	27.3	27.1	28.7	27.4
49	29.9	25.4	38.5	32.8	23.8	28.5	29.4	49.0	23.1	25.0	36.7	31.7	28.1	23.5	31.3
50	36.6	38.0	36.5	38.5	29.5	31.1	37.7	50.0	26.6	33.6	36.0	33.0	28.1	31.9	37.3
51	35.0	36.6	43.2	34.5	32.4	32.5	39.1	51.0	36.9	36.9	35.9	38.4	27.6	31.8	37.2
52	28.2	31.6	38.3	30.0	28.3	30.2	34.8	52.0	34.7	35.0	40.8	33.0	26.5	34.5	36.5
53	29.6	26.7	37.1	31.0	23.8	28.3	32.8	53.0	24.6	32.7	30.0	30.5	26.0	23.6	31.6
54	30.3	41.6	41.4	39.8	34.3	34.8	40.1	54.0	35.2	36.8	33.0	35.6	31.1	35.4	40.2
55	39.3	34.7	38.9	36.0	31.3	32.2	34.0	55.0	39.7	41.8	38.4	39.4	38.0	35.9	44.4
56	34.8	34.2	36.1	29.2	27.2	31.9	35.6	56.0	43.1	39.2	36.4	41.3	37.1	37.7	40.3
57	26.6	28.7	39.5	33.8	25.3	28.4	30.2	57.0	31.5	42.4	39.4	38.8	32.1	35.0	40.1
58	26.5	27.5	29.0	26.7	24.3	26.3	27.5	58.0	27.0	32.9	41.8	31.2	30.1	34.7	32.8
59	32.7	30.2	32.3	31.5	24.4	26.3	35.0	59.0	23.8	30.0	34.6	28.8	27.6	32.7	32.1
60	29.6	31.2	41.2	27.9	24.4	27.1	34.1	60.0	25.9	29.6	31.8	31.3	28.2	30.8	29.4
61	32.1	30.3	28.3	25.2	26.9	26.3	32.4	61.0	25.8	29.4	30.5	30.8	25.8	20.8	23.7
62	25.5	29.4	34.3	28.7	27.5	23.7	29.9	62.0	20.1	31.8	25.2	26.9	24.5	21.9	25.2
63	24.2	36.4	41.9	34.8	22.5	30.5	30.3	63.0	34.7	31.9	39.8	36.8	31.1	26.5	35.4
64	33.4	30.6	37.8	34.8	29.1	29.5	32.9	64.0	27.6	31.1	37.2	39.2	28.4	32.1	34.3
65	29.9	32.8	34.0	31.2	24.0	25.5	36.1	65.0	31.3	30.6	31.6	34.3	25.0	28.6	32.5
66	22.4	25.9	29.4	29.6	24.0	23.9	27.1	66.0	20.6	25.5	33.6	31.0	21.8	22.2	26.7
67	21.3	30.3	33.8	26.8	24.1	25.5	28.0	67.0	21.9	24.0	25.9	26.5	23.3	24.9	26.2
68	24.3	24.9	33.4	34.2	26.6	27.5	30.0	68.0	29.9	32.5	34.3	27.6	24.6	29.8	30.0
69	27.4	22.8	31.2	36.2	28.6	22.5	30.7	69.0	29.2	31.9	38.2	26.2	27.0	27.0	29.3
70	23.6	27.5	34.0	26.5	22.4	27.5	27.8	70.0	24.5	29.4	31.7	27.7	26.9	25.0	24.5
71	34.8	29.1	30.6	33.0	27.2	26.1	31.9	71.0	30.8	34.4	35.2	33.7	26.7	35.4	35.2
72	30.7	28.6	35.2	30.9	27.0	25.2	35.0	72.0	36.1	40.0	43.5	36.9	32.1	34.3	35.9
73	31.8	34.9	37.8	37.0	33.3	31.7	35.8	73.0	34.6	33.5	39.6	33.0	30.3	33.3	37.7
74	25.8	27.3	25.0	29.2	22.7	28.9	29.0	74.0	30.3	32.1	40.2	29.7	28.8	29.4	35.5
75	27.8	31.7	33.7	35.0	26.7	30.3	33.6	75.0	25.6	24.6	34.4	31.7	24.8	33.2	33.1
76	34.1	44.9	46.9	42.2	39.6	41.5	41.5	76.0	48.6	45.5	49.6	49.5	49.1	43.3	47.0
77	34.2	43.1	53.7	41.2	36.0	38.4	45.5	77.0	43.9	42.8	51.6	45.5	43.3	35.5	44.2

Supplementary Table 6. Comparison between number of water molecules detected in high resolution cryogenic data and RT data. The table shows the total number of water molecules detected in the whole structure and within 3.5 Å of each water channel. Number of water molecules in the channels between cryogenic data and RT data are quite comparable. However, water molecules detected in O1 Channel B are higher in cryogenic data than RT.

	4UB6	5B66	combined data(PDB ID 7RF1)
X-ray source	XFEL	synchrotron	XFEL
Temperature during collection	cryo	cryo	RT
Radiation	Damage free	0.03 MGy	Damage free
State	0F	0F	Combined data (0F-3F)
Resolution	1.95 Å	1.85 Å	1.89 Å
Reference	Suga et al.[13]	Tanaka et al.[14]	Current study
No. of water molecules in the structure	2624	4066	1960
No. of water molecules in O1 Channel A	19	20	20
No. of water molecules in O1 Channel B	23	28	18
No. of water molecules in O4 Channel	20	20	18
No. of water molecules in Cl1 Channel A	16	16	16
No. of water molecules in Cl1 Channel B	26	24	23
No. of water molecules in the channels	104	108	95
No. of water molecules in the channels within 5 Å from the bulk	25	22	16

Supplementary Table 7. Number of cryo-protectant or additive molecules detected in RT and cryo-structure data

PDB (ID)	Reference	State	Sample condition	Cryo-protectant/additives	Numbers of cryo-protectant molecules
6JLJ	Suga et al.[1]	0F	cryo	glycerol	20
4UB6	Suga et al.[13]	0F	cryo	glycerol	39
5WS5	Suga et al.[15]	0F	RT	glycerol	5
5B5E	Tanaka et al.[14]	0F	cryo	DMSO	112
5B66	Tanaka et al.[14]	0F	cryo	DMSO	97
3WU2	Umena et al.[7]	0F	cryo	glycerol	36

Supplementary References

1. Suga M*, et al.* An oxyl/oxo mechanism for oxygen-oxygen coupling in PSII revealed by an x-ray free-electron laser. *Science* **366**, 334-338 (2019).
2. Kern J*, et al.* Structures of the intermediates of Kok's photosynthetic water oxidation clock. *Nature* **563**, 421-425 (2018).
3. Chovancova E*, et al.* CAVER 3.0: a tool for the analysis of transport pathways in dynamic protein structures. *PLoS Comput. Biol.* **8**, e1002708 (2012).
4. Ho FM, Styring S. Access channels and methanol binding site to the $CaMn_4$ cluster in Photosystem II based on solvent accessibility simulations, with implications for substrate water access. *Biochim. Biophys. Acta* **1777**, 140-153 (2008).
5. Murray JW, Barber J. Structural characteristics of channels and pathways in photosystem II including the identification of an oxygen channel. *J. Struct. Biol.* **159**, 228-237 (2007).
6. Gabdulkhakov A*, et al.* Probing the accessibility of the Mn_4Ca cluster in photosystem II: channels calculation, noble gas derivatization, and cocrystallization with DMSO. *Structure* **17**, 1223-1234 (2009).
7. Umena Y, Kawakami K, Shen J-R, Kamiya N. Crystal structure of oxygen-evolving photosystem II at a resolution of 1.9 Å. *Nature* **473**, 55-60 (2011).
8. Vassiliev S, Zaraiskaya T, Bruce D. Exploring the energetics of water permeation in photosystem II by multiple steered molecular dynamics simulations. *Biochim. Biophys. Acta* **1817**, 1671-1678 (2012).
9. Ogata K, Yuki T, Hatakeyama M, Uchida W, Nakamura S. All-atom molecular dynamics simulation of photosystem II embedded in thylakoid membrane. *J Am Chem Soc* **135**, 15670-15673 (2013).
10. Sakashita N, Watanabe HC, Ikeda T, Saito K, Ishikita H. Origins of water molecules in the photosystem II crystal structure. *Biochemistry* **56**, 3049-3057 (2017).
11. Weisz DA, Gross ML, Pakrasi HB. Reactive oxygen species leave a damage trail that reveals water channels in Photosystem II. *Sci. Adv.* **3**, eaao3013 (2017).
12. Hattne J*, et al.* Accurate macromolecular structures using minimal measurements from X-ray free-electron lasers. *Nat Methods* **11**, 545-548 (2014).

13. Suga M, *et al.* Native structure of photosystem II at 1.95 Å resolution viewed by femtosecond X-ray pulses. *Nature* **517**, 99-103 (2015).
14. Tanaka A, Fukushima Y, Kamiya N. Two different structures of the oxygen-evolving complex in the same polypeptide frameworks of photosystem II. *J. Am. Chem. Soc.* **139**, 1718-1721 (2017).
15. Suga M, *et al.* Light-induced structural changes and the site of O=O bond formation in PSII caught by XFEL. *Nature* **543**, 131-135 (2017).
