# Supplementary material for: Structural dynamics in the water and proton channels of photosystem II during the S2 to S3 transition
Source: Nat Commun. 2021 Nov 11;12:6531. doi: 10.1038/s41467-021-26781-z (PMC8585918; doi:10.1038/s41467-021-26781-z)
Supplement: Supplementary file 3 — Reporting summary [file 41467_2021_26781_MOESM3_ESM.pdf]

nature research

Corresponding author(s): Jan Kern, Johannes Messinger, Athina Zouni, Junko Yano, Vittal Yachandra

Last updated by author(s): Oct 6, 2021

Reporting Summary

Nature Research wishes to improve the reproducibility of the work that we publish. This form provides structure for consistency and transparency in reporting. For further information on Nature Research policies, see our Editorial Policies and the Editorial Policy Checklist.

Statistics

For all statistical analyses, confirm that the following items are present in the figure legend, table legend, main text, or Methods section.

n/a	Confirmed	
☐	☒	The exact sample size (n) for each experimental group/condition, given as a discrete number and unit of measurement
☐	☒	A statement on whether measurements were taken from distinct samples or whether the same sample was measured repeatedly
☒	☐	The statistical test(s) used AND whether they are one- or two-sided<br>*Only common tests should be described solely by name; describe more complex techniques in the Methods section.*
☒	☐	A description of all covariates tested
☒	☐	A description of any assumptions or corrections, such as tests of normality and adjustment for multiple comparisons
☐	☒	A full description of the statistical parameters including central tendency (e.g. means) or other basic estimates (e.g. regression coefficient) AND variation (e.g. standard deviation) or associated estimates of uncertainty (e.g. confidence intervals)
☒	☐	For null hypothesis testing, the test statistic (e.g. F, t, r) with confidence intervals, effect sizes, degrees of freedom and P value noted<br>*Give P values as exact values whenever suitable.*
☒	☐	For Bayesian analysis, information on the choice of priors and Markov chain Monte Carlo settings
☒	☐	For hierarchical and complex designs, identification of the appropriate level for tests and full reporting of outcomes
☒	☐	Estimates of effect sizes (e.g. Cohen's d, Pearson's r), indicating how they were calculated

Our web collection on statistics for biologists contains articles on many of the points above.

Software and code

Policy information about availability of computer code

Data collection	We used the live version of psana (ana-current) provided by SLAC and development versions of open source programs dials.stills_process, the cctbx.xfel GUI and cxi.merge, which are distributed with DIALS packages publicly available at http://dials.github.io, for data collection.
Data analysis	We used development versions of open source programs dials.stills_process, the cctbx.xfel GUI and cxi.merge, which are distributed with DIALS packages publicly available at http://dials.github.io and END/RAPID structure factor modification software available at http://bl831.als.lbl.gov/END/RAPID/. We also used Phenix structure refinement software available at https://phenix-online.org (version 1.19.2-4158), the CCP4 package (version 7.1), Coot structure visualization and modification software version 0.8.8-pre, and PyMol structure visualization and figure rendering software version 2.4.

For manuscripts utilizing custom algorithms or software that are central to the research but not yet described in published literature, software must be made available to editors and reviewers. We strongly encourage code deposition in a community repository (e.g. GitHub). See the Nature Research guidelines for submitting code & software for further information.

Data

Policy information about availability of data

All manuscripts must include a data availability statement. This statement should provide the following information, where applicable:

- Accession codes, unique identifiers, or web links for publicly available datasets
- A list of figures that have associated raw data
- A description of any restrictions on data availability

The atomic coordinates and structure factors have been deposited in the Protein Data Bank, www.pdb.org (PDB ID code 7RF1 for the combined data , 7RF2 for the 0F, 7RF3 for the 1F, 7RF4 for the 2F(50s), 7RF5 for the 2F(150s), 7RF6 for the 2F(250s), 7RF7 for the 2F(400s), and 7RF8 for the 2F(200ms) data). Already published protein structures used in this study can be found in the Protein Data Bank under accession codes 3JCU, 3WU2, 4UB6, 5TIS and 6JLJ. Source data are

provided with this paper.

Field-specific reporting

Please select the one below that is the best fit for your research. If you are not sure, read the appropriate sections before making your selection.

☒ Life sciences ☐ Behavioural & social sciences ☐ Ecological, evolutionary & environmental sciences

For a reference copy of the document with all sections, see nature.com/documents/nr-reporting-summary-flat.pdf

Life sciences study design

All studies must disclose on these points even when the disclosure is negative.

Sample size	As this is a crystallographic experiment sample size is not considered relevant. The number of individual crystals used for all data sets is stated in the Method section and Supplementary Data Table 1
Data exclusions	Lattices not extending to 3 Ångstrom resolution were excluded. Lattices not belonging to the same unit cell were excluded using the unit cell outlier rejection mechanism in cxi.merge using a threshold of 1% for deviation from the reference.
Replication	Initial data was processed three times using different subsets of the experimental data to confirm main findings. Refinement was replicated 100 times using the END/RAPID approach described in the methods section and in previous publications (Ibrahim et al PNAS 2020, Lang et al PNAS 2014), confirming the results and giving an error estimate for atom positions of interest.
Randomization	Randomization is not relevant for this experiment. To reduce effects of systematic errors different illumination states were interleaved as much as possible during data collection within the constraints of the XFEL experiment.
Blinding	Investigators were not blinded to group allocation during data collection. Blinding was not relevant to this study as data for all different states studied was collected interleaved, ensuring similar experimental conditions. Subsequent data treatment was also performed in the same way for all data sets.

Reporting for specific materials, systems and methods

We require information from authors about some types of materials, experimental systems and methods used in many studies. Here, indicate whether each material, system or method listed is relevant to your study. If you are not sure if a list item applies to your research, read the appropriate section before selecting a response.

Materials & experimental systems

n/a	Involved in the study	
☒	☐	Antibodies
☒	☐	Eukaryotic cell lines
☒	☐	Palaeontology and archaeology
☒	☐	Animals and other organisms
☒	☐	Human research participants
☒	☐	Clinical data
☒	☐	Dual use research of concern

Methods

n/a	Involved in the study	
☒	☐	ChIP-seq
☒	☐	Flow cytometry
☒	☐	MRI-based neuroimaging
